# Expanding access to parasite-based malaria diagnosis through retail drug shops in Tanzania: evidence from a randomized trial and implications for treatment

**DOI:** 10.1186/s12936-016-1658-y

**Published:** 2017-01-03

**Authors:** Kathleen Maloney, Abigail Ward, Bonnie Krenz, Nora Petty, Lindsay Bryson, Caitlin Dolkart, Theodoor Visser, Arnaud Le Menach, Valerie K. Scott, Justin M. Cohen, Esther Mtumbuka, Sigsbert Mkude

**Affiliations:** 1 Clinton Health Access Initiative, Inc., 383 Dorchester Avenue Suite 400, Boston, MA 02127 USA; 2 Clinton Health Access Initiative, Inc., Tanzania Office, Dar es Salaam, Tanzania; 3 Tanzania National Malaria Control Programme, Dar es Salaam, Tanzania

**Keywords:** Parasite-based malaria diagnosis, Rapid diagnostic test, Private retail sector, Tanzania

## Abstract

**Background:**

Tanzania has seen a reduction in the fraction of fevers caused by malaria, likely due in part to scale-up of control measures. While national guidelines require parasite-based diagnosis prior to treatment, it is estimated that more than half of suspected malaria treatment-seeking in Tanzania initiates in the private retail sector, where diagnosis by malaria rapid diagnostic test (RDT) or microscopy is illegal. This pilot study investigated whether the introduction of RDTs into Accredited Drug Dispensing Outlets (ADDOs) under realistic market conditions would improve case management practices.

**Methods:**

Dispensers from ADDOs in two intervention districts in Tanzania were trained to stock and perform RDTs and monitored quarterly. Each district was assigned a different recommended retail price to evaluate the need for a subsidy. Malaria RDT and artemisinin-based combination therapy (ACT) uptake and availability were measured pre-intervention and 1 year post-intervention through structured surveys of ADDO owners and exiting customers in both intervention districts and one contiguous control district. Descriptive analysis and logistic regression were used to compare the three districts and identify predictive variables for testing.

**Results and discussion:**

A total of 310 dispensers from 262 ADDOs were trained to stock and perform RDTs. RDT availability in intervention ADDOs increased from 1% (n = 172) to 73% (n = 163) during the study; ACT medicines were available in 75% of 260 pre-intervention and 68% of 254 post-intervention ADDOs. Pre-treatment testing performed within the ADDO increased from 0 to 65% of suspected malaria patients who visited a shop (95% CI 60.8–69.6%) with no difference between intervention districts. Overall parasite-based diagnosis increased from 19 to 74% in intervention districts and from 3 to 18% in the control district. Prior knowledge of RDT availability (aOR = 1.9, p = 0.03) and RDT experience (aOR = 1.9, p = 0.01) were predictors for testing. Adherence data indicated that 75% of malaria positives received ACT, while 3% of negatives received ACT.

**Conclusions:**

Trained and supervised ADDO dispensers in rural Tanzania performed and sold RDTs under real market conditions to two-thirds of suspected malaria patients during this one-year pilot. These results support the hypothesis that introducing RDTs into regulated private retail sector settings can improve malaria testing and treatment practices without an RDT subsidy.

*Trial registration* ISRCTN ISRCTN14115509

## Background

Malaria prevalence among children under 5 years old in Tanzania declined from 18% in 2007 to 9% in 2011, whereas 2-week fever prevalence remained approximately 20% over the same time period, from 19 to 20% [1, 2]. This reduction in the proportion of fevers caused by malaria is likely due at least in part to Tanzania’s recent scale-up of prevention and treatment measures [3–5]. Presumptive treatment of fevers with artemisinin-based combination therapy (ACT) thus will increasingly result in incorrect malaria diagnoses, prescription and wastage of inappropriate medications, and subsequent delays in obtaining effective treatment for the true cause of illness [6–9].

The Tanzania National Malaria Control Programme (NMCP) case management guidelines require suspected malaria cases to receive parasite-based diagnosis by microscopy or rapid diagnostic test (RDT) prior to treatment with anti-malarial drugs [10, 11]. In practice, however, up to 54% of those seeking treatment for suspected malaria in Tanzania first visit the private retail sector, where malaria diagnostics are currently prohibited from being sold and administered [12]. As a result, parasite-based testing is not received by many suspected malaria patients.

The Tanzanian private retail sector includes both unregistered outlets and a network of more than 6000 registered shops or Accredited Drug Dispensing Outlets (ADDOs) (*duka la dawa muhimu* or *DLDM* in Kiswahili) regulated by the Pharmacy Council. Unlike unregistered outlets, ADDOs are permitted to stock and sell both over-the-counter medicines and certain classes of prescription medications, including ACT [13–15]. To obtain a Pharmacy Council permit each year, ADDOs owners must meet certain conditions related to the premises, training and certification of a dispenser who may or may not be the owner, and the products stocked. Like all other private retail outlets, RDTs may not be sold or performed at ADDOs, however.

Malaria RDTs have been safely administered by non-medical personnel in several previous settings [16–18]. The introduction of RDTs into ADDOs has the potential to improve fever case management by increasing availability, access, and use of parasite-based diagnosis. It is unclear, however, whether customers will be willing to pay the extra cost for diagnosis, whether ADDO owners would encourage RDT use, whether RDTs might require subsidization to encourage uptake in the private sector, and whether treatment choices would adhere to test results. To investigate these questions, this pilot evaluated the operational feasibility of selling RDTs through ADDOs and measured changes in suspected malaria patient case management that occurred as a result of making RDTs available at two different prices.

## Methods

### Study area and population

**Fig. 1:**
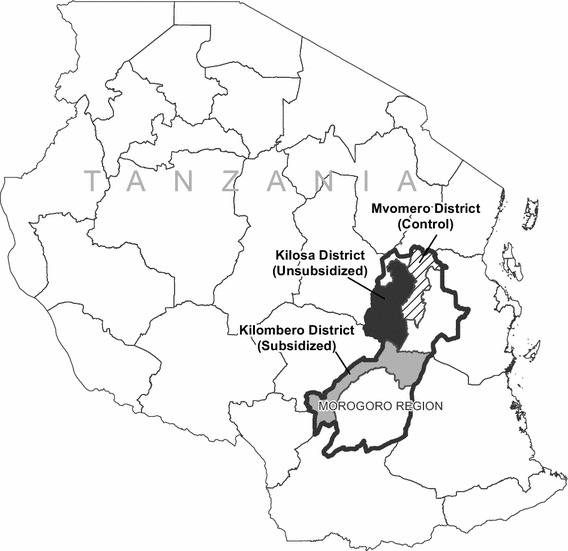
Map of the study area

This pilot was conceived and designed in partnership with the NMCP and Pharmacy Council, with a primary objective of informing national policymaking on legalizing the stocking and performance of RDTs through ADDOs. The study was conducted in three districts in Morogoro Region: Kilombero, Kilosa, and Mvomero. These three districts were selected due to their high density of ADDOs, moderate *Plasmodium falciparum* prevalence compared with national data (13% in Morogoro Region [2]), and convenient proximity to Dar es Salaam. The three districts are mostly rural, with a total estimated population of 1.7 million people in 2012 [19]. Peak malaria incidence corresponds to the two rainy seasons, one between March and May and the other between September and December. Kilombero and Kilosa were assigned via random number generator as the two intervention areas where ADDO dispensers were trained to stock, sell, and administer RDTs, and Mvomero served as the control area (Fig. 1).

### Intervention design

All licensed ADDO dispensers currently working in an ADDO in the two intervention areas were invited to participate in the study through the District Malaria Focal Person, who is responsible for overseeing local malaria-related activities under the leadership of the District Medical Officer. In April–May 2013, six two-day trainings were held in each intervention district, each led by a national-level trainer and the district malaria focal person. Trainings covered signs and symptoms of uncomplicated and severe malaria, stocking, use, and disposal of RDTs, and appropriate case management based on RDT results. Dispensers were instructed to prescribe Tanzania’s first-line treatment, artemether + lumefantrine (commonly referred to as ALu in Tanzania), to test-positive customers based on an ALu dosing reference chart provided to the dispensers. Dispensers were trained to refer customers with signs and symptoms of severe illness, suspected malaria patients who tested negative for malaria, and suspected malaria patients whose illness did not improve within 48 h to the nearest public health facility along with the results of their malaria test.

Upon passing both practical and written evaluations, certified RDT dispensers were provided with an ID badge granting permission to perform RDTs in their ADDO, a sharps box, an RDT performance and case management job aid, and a weather-proof storefront sign advertising that malaria testing was available. Permission was also granted for the ADDO dispenser to prescribe an ALu to all patients who tested positive for malaria.

To mimic real market conditions, existing supply chains were utilized: ADDOs in the two districts were instructed to purchase ParaHIT® Ag Pf RDTs from seven wholesalers. The wholesalers, in turn, were instructed to purchase RDTs from an importer in Dar es Salaam, who purchased the RDTs directly from the manufacturer for a fixed price pre-negotiated by the research team. Importers, wholesalers and ADDOs all agreed to fixed mark ups.

ADDOs in Kilosa were asked to sell RDTs for a recommended retail price (RRP) of 1100 Tanzanian Shillings (USD $0.67 in May 2013) each, based on willingness-to-pay responses from pre-intervention exit interviews, analysis of markups in analogous commodities, and price negotiation. In Kilombero, ADDOs were asked to sell RDTs for 50% less or 500 Tanzanian Shillings (USD $0.32 in May 2013) [20]. To enable the ADDOs in Kilombero to charge the lower price, the research team asked the importer to sell RDTs to wholesalers in Kilombero at a 50% discount. On a monthly basis, the importer received financial compensation from the research team equal to the total discount provided to wholesalers located in Kilombero. The research team monitored stock levels at the importer level to prevent stock outs.

Gloves were included in the RDT boxes. A unique blue-and-white “mRDT” checkmark logo was placed on ParaHIT® boxes, ID badges, job aids, and storefront signs in order to build brand recognition.

Trained study staff provided supportive supervision during quarterly monitoring visits. At each visit, the certified dispenser at each participating ADDO was observed performing an RDT on a patient and evaluated according to a 17-point checklist adapted from the WHO “Checklist for direct observation of health workers performing Rapid Diagnostic Tests (RDT) for malaria” [21]. The supervisors also reported on stocking safety, shop hygiene, and waste disposal practices [22].

### Study design

The impact of the intervention was assessed by comparing the change in availability and use of RDTs at ADDOs in the intervention districts with those in the control district, Mvomero, where ADDOs were not given access to the RDTs, training, or supervision.

#### Outlet surveys

RDT and ACT availability were measured through two cross-sectional ADDO surveys, one conducted prior to the dispenser training in March 2013 and the second a year after the 2013 training, in May 2014. All ADDOs were eligible for selection for the pre-intervention survey, while only ADDOs with certified dispensers were eligible to participate in the post-intervention survey of the intervention districts. Ninety-one ADDOs per district were selected using random number generation based on sample-size calculations to ensure 80% power. The sample size is assumed to be sufficient to detect a five percentage-point difference in ADDO RDT availability.

A Kiswahili or English-version structured questionnaire was used during the face-to-face interviews with ADDO dispensers depending on the language preference of the respondent. The primary outcome was RDT availability, defined as the proportion of ADDOs with RDTs in stock on the day of the survey and a trained, certified dispenser present to administer the test. A secondary outcome was ACT availability, defined as the proportion of ADDOs that reported having ACT medicines in stock for the 30 days prior to the survey.

#### Customer exit interviews

Face-to-face exit interviews were conducted prior to the dispenser training (March 2013) and a year after the intervention began (May 2014) using a structured questionnaire in Kiswahili. Customers eligible for the exit interview were at least 18 years old and either seeking treatment in the ADDO for fever or suspected malaria or attempting to purchase an anti-malarial for themselves or someone else (the “patient”). A sample size of 400 eligible customers per district was estimated based on two-tailed sample size calculations designed to detect a 5%-point difference in RDT availability or 80% power. Pre-intervention, 1–3 customers were interviewed at each sampled ADDO. The sample size was halved for the pre-intervention survey based on very low predicted availability of RDTs and a primary goal of comparing similarity between districts. Post-intervention, enumerators attempted to survey all eligible customers during one full day at the ADDO in order to self-weight traffic variation between shops.

If the customer and the patient were not the same person, demographic data were collected on both the customer and the patient, and illness data were collected about the patient. Customers were asked whether patients (either themselves or others) had received diagnostic testing at the ADDO, had been previously tested elsewhere, how much they had paid for RDTs at the ADDO, and whether they had purchased anti-malarial treatment.

#### Statistical analysis

Chi square tests were used to compare differences before and after intervention and between intervention and control districts in (a) the proportion of patients seeking treatment at ADDOs who received a parasite-based diagnostic test, (b) the median price paid for an RDT in an ADDO, (c) the proportion of patients who received a parasite-based diagnosis, (d) adherence to test results in terms of receiving ACT when testing positive and not receiving one when testing negative, and (e) the proportion of test-negative patients who received an antibiotic.

Logistic regression models were then used to identify factors associated with receiving an RDT in an ADDO or receiving a parasite-based diagnosis anywhere, with shop included as a random effect to account for repeated sampling of visits to the same ADDOs. Tested covariates included study district and survey, demographic variables (customer and patient gender, age, completed education, and wealth quintile) as well as RDT knowledge and practice indicators regarding previous experience and attitudes about testing. Wealth quintiles were derived from a wealth index based on a set of household asset questions estimated using principal components analysis using similar methodology to Demographic and Health Surveys [23]. Covariates with p < 0.2 in bivariable models were included in multivariable models. Logistic regression models were also used to compare odds of treatment outcomes controlling for study district, survey, parasite-based diagnosis, and test result.

## Results

Ninety-five percent of ADDO dispensers passed the 2-day training. In total, 310 dispensers from 262 ADDOs, 164 (147 ADDOs) from Kilombero in the lower-priced or “subsidized” district and 146 (115 ADDOs) from Kilosa the higher priced or “unsubsidized” district, were certified and allowed to sell and perform RDTs. The number of outlet surveys and exit interviews completed are presented in Table 1.

**Table 1:** Total shop and exit interviews conducted and included in analysis

District	Pre-intervention (March 2013)				Post-intervention (May 2014)			
	ADDOs surveyed	ADDOs with ACT in Stock^a^	Total Exit Interviews	Interviews where patient present and ACT in Stock^b^	ADDOs surveyed	ADDOs with ACT in Stock^a^	Total Exit Interviews	Interviews where patient present and ACT in Stock^b^
Subsidized (Kilombero)	87	59	91	54	76	52	266	217
Unsubsidized (Kilosa)	85	66	82	46	87	63	330	247
Control (Mvomero)	88	69	86	59	91	57	359	244
Total	260	194	259	159	254	172	955	708

^a^’ACT in stock’ defined as no stock-outs in the past 30 days

^b^Only customer interviews from shops with ACT medicines in stock and the patient present were included in analysis (i.e., the patient had the opportunity to be tested and purchase an ACT medicine)

### Diagnostic and treatment availability in ADDOs

Pre-intervention outlet surveys found that RDTs were not available in any surveyed ADDOs. Post-intervention, 73% of ADDOs in intervention districts had RDTs in stock and an RDT-certified dispenser present. The proportion of ADDOs with a certified dispenser and RDTs available was higher in the “unsubsidized” district Kilosa (85%, 95% CI 78–92%) than the “subsidized” district Kilombero (64%, 95% CI 55–72%, p < 0.001). Across the study districts, the proportion of ADDOs with ACTs available was 75% pre-intervention and 68% post-intervention. ACT availability did not differ significantly between districts or pre- or post-intervention (p > 0.05 for all).

### Customer and patient characteristics

A total of 1214 customers seeking treatment for suspected malaria, either for themselves or someone else, were interviewed: 259 pre-intervention and 955 post-intervention (Table 1). Demographic characteristics of customers were generally similar between districts during each survey (Table 2). The proportion of exit interviews in which the patient was present at the ADDO was significantly higher in intervention districts post-intervention. ADDOs were the first place treatment was sought for 75% of patient visits across surveys and districts (Table 3).

**Table 2:** Characteristics of anti-malarial customers

	Pre-intervention				Post-intervention			
	Subsidized (n = 91)	Unsubsidized (n = 82)	Control (n = 86)	*χ* ^2^ p value	Subsidized (n = 266)	Unsubsidized (n = 330)	Control (n = 359)	*χ* ^2^ p value
Customer age (median, range)	30 (18–67)	34 (18–68)	33 (18–83)		30 (6–79)	32 (12–92)	30 (14–82)	
Male (%, 95% CI)	44.0 (33.7–54.3)	47.6 (36.6–58.5)	52.3 (41.7–63.0)	0.536	47.4 (41.3–53.4)	49.4 (44.0–54.8)	51.5 (46.3–56.7)	0.585
Completed > Primary school (%, 95% CI)	15.4 (7.9–22.9)	24.4 (15.0–33.8)	20.9 (12.2–29.6)	0.327	21.1 (16.1–26.0)	13.9 (10.2–17.7)	21.2 (16.9–25.4)	0.025
Wealth quintile (%, 95% CI)								
Lowest	14.3 (7.0–21.5)	30.5 (20.4–40.6)	17.4 (9.3–25.5)	0.337	17.7 (13.1–22.3)	18.2 (14.1–22.4)	24.8 (20.3–29.3)	0.248
Lower	20.9 (12.4–29.3)	18.3 (9.8–26.8)	20.9 (12.2–29.6)		21.8 (16.8–26.8)	21.9 (17.4–26.4)	18.1 (14.1–22.1)	
Middle	23.1 (14.3–31.8)	13.4 (6.0–20.9)	22.1 (13.2–31.0)		22.9 (17.9–28.0)	18.2 (14.1–22.4)	20.6 (16.4–24.8)	
Higher	20.9 (12.4–29.3)	20.7 (11.9–29.6)	20.9 (12.2–29.6)		20.3 (15.5–25.1)	21.3 (16.8–25.7)	17.5 (13.6–21.5)	
Highest	20.9 (12.4–29.3)	17.1 (8.8–25.3)	18.6 (10.3–26.9)		17.3 (12.7–21.9)	20.4 (16.0–24.7)	18.9 (14.9–23.0)	
Patient is customer or is with customer (present at ADDO) (%, 95% CI)	59.3 (49.1–69.5)	59.1 (45.2–67.0)	68.6 (58.7–78.5)	0.221	81.6 (76.9–86.3)	74.8 (70.2–79.5)	68.0 (63.1–72.8)	0.001

**Table 3:** Characteristics of patients present at the ADDO

	Pre-intervention				Post-intervention			
	Subsidized (n = 54)	Unsubsidized (n = 46)	Control (n = 59)	*χ* ^2^ p value	Subsidized (n = 217)	Unsubsidized (n = 247)	Control (n = 244)	*χ* ^2^ p value
Patient age (median, range)	24.5 (1–66)	22 (<1–62)	27 (<1–83)		23 (<1–79)	22 (1–92)	24 (1–74)	
Male (%, 95% CI)	48.1 (34.6–61.7)	47.8 (33.1–62.5)	50.8 (37.8–63.8)	0.941	47.9 (41.2–54.6)	53.0 (46.8–59.3)	50.8 (44.5–57.1)	0.546
Days Ill (median, range)	3 (<1, 14)	3 (<1, 30)	3 (<1, 14)		2 (<1, 60)	3 (<1, 30)	2 (<1, 60)	
ADDO first place treatment sought (%, 95% CI)	70.4 (58.0–82.8)	65.2 (51.2–79.2)	80.0 (69.2–90.1)	0.240	77.4 (71.8–83.0)	74.8 (69.4–80.2)	75.4 (70.0–80.8)	0.792
Malaria test prior to ADDO Visit (%, 95% CI)	16.7 (6.6–26.8)	21.7 (9.6–33.9)	3.4 (0.0–8.1)	0.015	11.1 (6.8–15.4)	13.4 (9.0–17.8)	14.9 (10.2–19.7)	0.502

### Diagnostic uptake

**Fig. 2:**
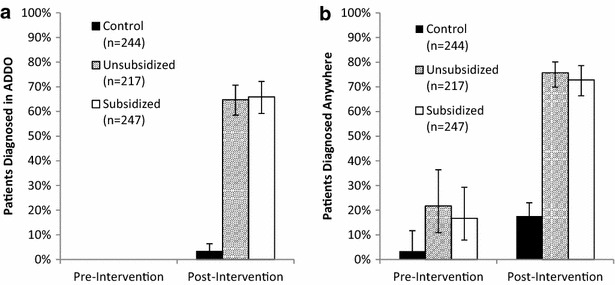
**a** Proportion of patients present at the ADDO who received a parasite-based diagnosis before and after intervention (p < 0.001 for changes in intervention districts; p = 0.159 for the control district). **b** Proportion of patients present in the ADDO reporting receiving parasite-based diagnostics at any location (p < 0.05 for pre- to post-intervention changes within each district)

Pre-intervention, no patients present in the ADDOs reported receiving a parasite-based test in an ADDO in any district. Post-intervention, 65% of patients present at ADDOs in the intervention districts (95% CI 60.8–69.6%) and 3% in the control district received an RDT (Fig. 2a). There was no difference in the fraction of eligible patients who were tested between the subsidized (66%, 95% CI 59–72%) and unsubsidized districts (67%, 95% CI 58–71%), (*χ* ^*2*^ < 0.1, p = 0.8).

Overall diagnostic uptake (in ADDOs or elsewhere prior to the ADDO visit) improved significantly in the intervention and control groups during the study (Fig. 2b). The proportion of patients present at the ADDO who received a diagnostic test increased from 19% pre-intervention to 74% post-intervention in the intervention districts and from 3 to 18% in the control district (p < 0.01 for both groups). The relative changes in testing were not significantly different between the intervention and control districts (OR = 2.5, p = 0.271).

Median prices for RDTs post-intervention reported by customers were 1100 Tanzanian Shillings in Kilosa, the unsubsidized district, and 500 Tanzanian Shillings in Kilombero, the subsidized district. There was no variance in median price from the recommended retail price in either district. No difference in RDT uptake was observed between the unsubsidized and subsidized districts (OR = 1.1, p = 0.868).

Patient knowledge and practices were associated with RDT purchasing (Table 4). The odds of purchasing an RDT were significantly higher for patients who had previous experience taking an RDT or were already aware that RDTs were available before coming to the ADDO, while patients who had sought treatment elsewhere before visiting the ADDO were significantly less likely to purchase an RDT.

**Table 4:** Factors associated with purchasing an RDT in the ADDO when the patient was present

Outcome: receiving an RDT in an ADDO if present to be tested (n = 464)	Bivariable			Multivariable		
	OR	95% CI	p value	OR	95% CI	p value
Intervention						
No subsidy	Reference					
Subsidy	1.1	(0.6–1.9)	0.868			
Customer gender (if not patient)						
Female	Reference					
Male	1.2	(0.5–2.7)	0.654			
Patient gender						
Female	Reference					
Male	1.0	(0.7–1.4)	0.920			
Customer age (if not patient)	1.0	(1.0–1.0)	0.198			
Patient age						
<5 years	Reference					
5− <14 years	1.5	(0.7–3.5)	0.332			
14+ years	0.7	(0.3–1.3)	0.254			
Customer education						
Primary	Reference					
Secondary and above	1.0	(0.6–1.6)	0.909			
Wealth index						
Lowest	Reference					
Lower	1.0	(0.5–2.0)	0.925			
Middle	0.9	(0.4–1.8)	0.723			
Higher	1.2	(0.6–2.5)	0.559			
Highest	1.0	(0.5–2.2)	0.943			
Has heard of RDTs	3.1	(2.0–4.8)	<0.001	1.7	(0.9–3.1)	0.088
Has taken an RDT before	3.1	(2.1–4.7)	<0.001	1.9	(1.2–3.0)	0.005
Sought treatment prior to ADDO visit	0.3	(0.2–0.5)	<0.001	0.3	(0.2–0.5)	<0.001
Knew before coming that testing was available in ADDOs	3.0	(2.0–4.7)	<0.001	1.9	(1.1–3.3)	0.032
Believes febrile patients should be blood tested before treating						
Never	Reference					
Sometimes	1.1	(0.1–8.9)	0.925			
Always	2.0	(0.3–13.9)	0.474			

### Treatment decisions

**Fig. 3:**
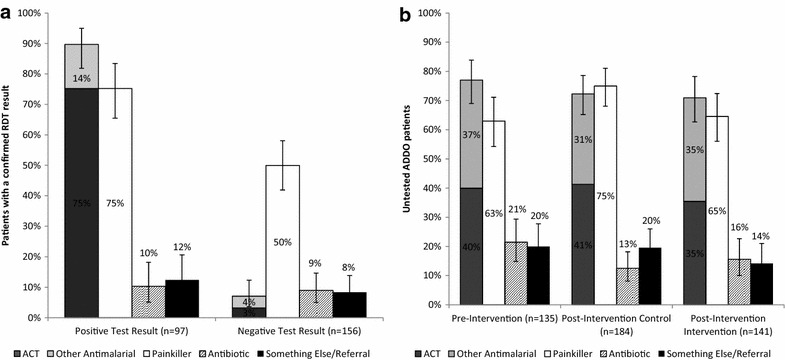
**a** Medications purchased by patients tested in ADDOs (intervention districts combined). **b** Medications purchased by untested ADDO patients. Percentages are not mutually exclusive; patients may have received more than one treatment type

The overall proportion of patients who received an ACT increased from 40 to 43% in the control district and decreased from 40 to 32% in the intervention districts, although these changes were not significantly different after controlling for other factors (OR = 0.6, p = 0.294). Forty-one percent of patients who received an RDT tested positive for malaria across the intervention districts (95% CI 35.7–46.8%). Among patients with a positive test, 90% (95% CI 81.9–94.9%) received an anti-malarial, and 75% (95% CI 65.5–83.5%) received ACT. Among customers testing negative, 7% (95% CI 3.6–12.3%) received an anti-malarial (Fig. 3a). There was no significant difference in the proportion of those who did not receive an RDT who purchased an anti-malarial (35% in intervention districts, 41% in control) (Fig. 3b).

The proportion of patients who received an antibiotic did not change significantly comparing intervention and control groups from pre- to post-intervention (OR = 0.9, p = 0.837). Post-intervention, the proportion of patients purchasing antibiotics also did not vary significantly comparing those who tested positive with those who tested negative (10% vs 9%, p = 0.73) (Fig. 3a).

## Discussion

The results from this study suggest that ADDOs offer an important opportunity for improving malaria case management in Tanzania towards the national goal of 80% parasite-based diagnosis of suspected malaria patients. Two-thirds of suspected malaria patients who sought treatment at ADDOs with RDT-certified dispensers purchased RDTs. Improved testing rates through ADDOs in this pilot project resulted in improved targeting of ACTs to patients with malaria, with 90% of malaria-positive and 93% of malaria-negative ADDO-tested patients making appropriate treatment decisions according to their test results.

These results add to a growing body of evidence that malaria case management can be performed well outside of the formal public health sector. A pilot in Ghana that measured vendor-reported adherence to test results after the introduction of RDTs to 28 Licensed Chemical Sellers (private retail sector shops) found 89% of positives and 3% of negatives received ACT [24]. Vendors of 59 licensed drug shops in Uganda also reported high uptake (98%) and favorable adherence to test results (ALu was prescribed to 98% of positives and 1% of negatives) in a small-scale pilot study [25]. The higher uptake observed in these studies compared with this one may be attributable to self-reporting by drug shop vendors compared with customer interviews, a lower $0.20 price point (Uganda) compared to $0.31–0.67 in Tanzania, and/or variance in length of training and frequency of supervision. Not all studies have found such positive outcomes, however: a different study in Uganda with 92 shops selling RDTs that measured adherence through household surveys found only 32% of positives purchased an ACT medicine (7% of negatives) at a median $0.40 per test [16].

This pilot was successful in improving case management practices for several reasons. First, ADDOs are a highly utilized source for anti-malarials in Tanzania: ADDOs were the first place treatment was sought for 75% of interviewed patients, emphasizing the need for access to parasite-based diagnosis at this point of care. Second, nearly all ADDOs in the study area participated in the trainings and RDT program. ADDOs were kept engaged in the study through quarterly supervision and monitoring visits, which also allowed for frequent quality assessments on safety protocol. Third, proximity of the region to Dar es Salaam and relatively good road infrastructure allowed for existing supply chains to be utilized, creating a more realistic market scenario. Finally, negotiations with RDT suppliers and agreements with wholesalers and ADDOs on mark ups, ensured affordable recommended retail prices and enabled comparison of price points.

These results provided data to help guide several open questions surrounding an introduction of RDTs to the private retail market. A similar proportion of patients were willing to pay the higher ‘unsubsidized’ price of a 1100 Tanzanian Shillings or USD $0.67 for a RDT compared to the lower unsubsidized price of 500 Tanzania Shillings or USD $0.32 for the subsidized RDT, indicating that a subsidy may not be necessary to encourage testing uptake. Additionally, similar to the Ghana pilot [24], an increase in the purchase of antibiotics among those receiving a negative test result was not observed in this study.

The study was also subject to several limitations. A significant pre-intervention difference in parasite-based diagnostic rates between intervention (19%) and control (3%) districts suggests inherent differences in health facility testing uptake that may confound the effect of the intervention. A second limitation was the unforeseen mobility of ADDO dispensers, periodically changing jobs work in different ADDOs during the study period. While dispensers were certified in this study, it was unclear if the certification extended to the ADDO when the dispenser was not present. Dispenser movement complicated follow-up and suggests future consideration for specifying that dispensers and ADDOs be certified, and requiring ADDOs that sell RDTs to have a certified dispenser present. Additional follow-up challenges arose when ADDOs closed temporarily or permanently, or were not reachable for stock delivery or enumeration due to flooded roads and bridges. Also, although dispensers were trained to refer severe and negative cases to the nearest health facility, this study was not resourced to follow the outcomes of these patients. In practice, it is unlikely that all negative cases will complete the referral pathway, underscoring the importance of including diagnosis and treatment of non-malaria febrile illness and recognition of danger signs in ADDO dispenser training. Lastly, the post-intervention survey sampled from ADDOs that participated in the training in the intervention areas, and while these were the majority of the ADDOs in these districts, they are not representative of all ADDOs in the study area.

As Tanzania uses the results of this study to inform policy decisions, it will be important to consider how results might vary in different endemic settings and in more urban areas. Expansion of this program will also require evaluation of how best to ensure long-term routine supervision and training, as well as waste management for sharps and cassettes. Finally, a critical challenge to scale-up will be integration of the private retail sector into the routine surveillance system for capturing malaria case data. This information will be imperative for strategic decision-making as malaria prevalence declines across the country.

## Conclusions

This pilot study contributes to a growing evidence base that introducing RDTs to the private retail sector in low resource settings can increase parasite-based diagnostic rates for malaria and adherence to test results when dispensers are trained and supervised. The pilot study also showed that RDTs can be introduced under real market conditions; utilizing existing supply chains without subsidizing the cost of the RDT. While the intervention piloted here was limited to a rural setting in Tanzania’s specialized ADDO network, it provides a basis for policy decisions on scaling up RDT access across Tanzania to increase parasite-based diagnosis and rational use of ACT through heavily used existing channels. The results presented here support the goals for Tanzania’s National Strategic Plan and may have applications in similar settings.

## Abbreviations

ITN: insecticide-treated bed net
ACT: artemisinin-based combination therapy
RDT: rapid diagnostic test
NMCP: National Malaria Control Programme
WHO: World Health Organization
ADDO: Accredited Drug Dispensing Outlet
DLDM: duka la dawa muhimu
PC: Pharmacy Council
TFDA: Tanzania Food and Drug Administration
PDA: personal digital assistant
NIMR: National Institute for Medical Research
RRP: recommended retail price

## References

[1] Tanzania Commission for AIDS (TACAIDS), Zanzibar AIDS Commission (ZAC), National Bureau of Statistics (NBS), Office of Chief Government Statistician (OCGS), and Macro International Inc. Tanzania HIV/AIDS and Malaria Indicator Survey 2007–2008. 2008. http://dhsprogram.com/pubs/pdf/AIS6/AIS6_05_14_09.pdf. Accessed 2 Feb 2016.

[2] Tanzania Commission for AIDS (TACAIDS), Zanzibar AIDS Commission (ZAC), National Bureau of Statistics (NBS), Office of Chief Government Statistician (OCGS), and ICF International. Tanzania HIV/AIDS and Malaria Indicator Survey 2011–2012. 2013. http://dhsprogram.com/pubs/pdf/AIS11/AIS11.pdf. Accessed 2 Feb 2016.

[3] Bhattarai A, Ali AS, Kachur SP, Mårtensson A, Abbas AK, Khatib R (2007). Impact of artemisinin-based combination therapy and insecticide-treated nets on malaria burden in Zanzibar. PLoS Med..

[4] Steketee RW, Campbell CC (2010). Impact of national malaria control scale-up programmes in Africa: magnitude and attribution of effects. Malar J..

[5] Bhatt S, Weiss DJ, Cameron E, Bisanzio D, Mappin B, Dalrymple U (2015). The effect of malaria control on *Plasmodium falciparum* in Africa between 2000 and 2015. Nature.

[6] Whitty CJ, Chandler C, Ansah E, Leslie T, Staedke SG (2008). Deployment of ACT antimalarials for treatment of malaria: challenges and opportunities. Malar J..

[7] Reyburn H, Mbatia R, Drakeley C, Carneiro I, Mwakasungula E, Mwerinde O (2004). Overdiagnosis of malaria in patients with severe febrile illness in Tanzania: a prospective study. BMJ.

[8] Bloland P. Drug Resistance in malaria. World Health Organization. 2001. http://www.who.int/csr/resources/publications/drugresist/malaria.pdf?ua=1. Accessed 10 Dec 2014.

[9] Perkins MD, Bell DR (2008). Working without a blindfold: the critical role of diagnostics in malaria control. Malar J..

[10] Tanzania National Malaria Control Programme. Tanzania National Malaria Strategic Plan 2014–2020. The United Republic of Tanzania Ministry of Health and Social Welfare. 2014. http://ihi.eprints.org//3314/1/Malaria_Strategic_Plan_Full_Version_02_27_14.pdf. Accessed 23 Dec 2015.

[11] WHO. Guidelines for the treatment of malaria—2nd edition. Geneva: World Health Organization, 2010. http://www.afro.who.int/index.php?option=com_docman&task=doc_download&gid=5290. Accessed 3 Sept 2015.

[12] Thomson R, Festo C, Johanes B, Kalolella A, Bruxvoort K, Nchimbi H (2014). Has Tanzania embraced the green leaf? Results from outlet and household surveys before and after implementation of the affordable medicines facility-malaria. PLoS ONE.

[13] Ringsted FM, Massawe IS, Lemnge MM, Bygbjerg IC (2011). Saleability of anti-malarials in private drug shops in Muheza, Tanzania: a baseline study in an era of assumed artemisinin combination therapy (ACT). Malar J..

[14] Hetzel MW, Obrist B, Lengeler C, Msechu JJ, Nathan R, Dillip A (2008). Obstacles to prompt and effective malaria treatment lead to low community-coverage in two rural districts of Tanzania. BMC Public Health..

[15] Goodman C, Kachur SP, Abdulla S, Bloland P, Mills A (2009). Concentration and drug prices in the retail market for malaria treatment in rural Tanzania. Health Econ.

[16] Cohen J, Fink G, Berg K, Aber F, Jordan M, Maloney K (2012). Feasibility of Distributing rapid diagnostic tests for malaria in the retail sector: evidence from an implementation study in Uganda. PLoS ONE.

[17] Mukanga D, Babirye R, Peterson S, Pariyo GW, Ojiambo G, Tibenderana JK (2011). Can lay community health workers be trained to use diagnostics to distinguish and treat malaria and pneumonia in children? Lessons from rural Uganda. Trop Med Int Health..

[18] Mubi M, Janson A, Warsame M, Mårtensson A, Källander K, Petzold MG (2011). Malaria rapid testing by community health workers is effective and safe for targeting malaria treatment: randomised cross-over trial in Tanzania. PLoS ONE.

[19] 2012 Population and housing census of Tanzania. Tanzania National Bureau of Statistics. 2013. http://www.nbs.go.tz. Accessed 10 Dec 2014.

[20] Current and historical rate tables. XE currency converter. 2015. http://www.xe.com. Accessed 18 Dec 2015.

[21] World Health Organization. Universal access to malaria diagnostic testing: an operational manual. 2011. http://apps.who.int/iris/bitstream/10665/44657/1/9789241502092_eng.pdf. Accessed 17 Dec 2014.

[22] Ward A, Krenz B, Maloney K, Dolkart CF, Le Menach A, Cohen JM, et al. Dispenser performance administering malaria rapid diagnostic tests in the private retail sector of Tanzania. Poster presented at: 63rd American Society of Tropical Medicine & Hygiene Annual Meeting. 2014.

[23] Rutstein SO, Johnson K. The DHS Wealth Index. Calverton: ORC Macro; 2004. http://www.dhsprogram.com/pubs/pdf/CR6/CR6.pdf. Accessed 22 Dec 2015.

[24] Ansah EK, Narh-Bana S, Affran-Bonful H, Bart-Plange C, Cundill B, Gyapong M (2015). The impact of providing rapid diagnostic malaria tests on fever management in the private retail sector in Ghana: a cluster randomized trial. BMJ.

[25] Mbonye AK, Magnussen P, Lal S, Hansen KS, Cundill B, Chandler C (2015). A cluster randomised trial introducing rapid diagnostic tests into registered drug shops in Uganda: impact on appropriate treatment of malaria. PLoS ONE.

